# Comparison of ultrasonographic, radiographic and intra-operative findings in severe hip osteoarthritis

**DOI:** 10.1038/s41598-020-78235-z

**Published:** 2020-12-03

**Authors:** Mika T. Nevalainen, Kyösti V. Kauppinen, Tuukka Niinimäki, Simo S. Saarakkala

**Affiliations:** 1 Department of Diagnostic Radiology, Oulu University Hospital, P.O. Box 50, 90029 Oulu, Finland; 2 Medical Research Center Oulu, University of Oulu, P.O. Box 8000, 90029 Oulu, Finland; 3 Research Unit of Medical Imaging, Physics and Technology, Faculty of Medicine, University of Oulu, P.O. Box 5000, 90014 Oulu, Finland; 4 Department of Diagnostic Radiology, Turku University Hospital, P.O. Box 52, 20521 Turku, Finland; 5 Department of Surgery, Oulu University Hospital, P.O. Box 50, 90029 Oulu, Finland

**Keywords:** Musculoskeletal system, Rheumatic diseases, Musculoskeletal system, Osteoarthritis

## Abstract

Aim of this study was to assess the US findings of patients with late-stage hip OA undergoing total hip arthroplasty (THA), and to associate the US findings with conventional radiography (CR) and intraoperative findings. Moreover, the inter-rater reliability of hip US, and association between the US and Oxford Hip Score (OHS) were evaluated. Sixty-eight hips were included, and intraoperative findings were available on 48 hips. Mean patient age was 67.6 years and 38% were males. OA findings—osteophytes at femoral collum and anterosuperior acetabulum, femoral head deformity and effusion—were assessed on US, CR and THA. The diagnostic performance of US and CR was compared by applying the THA findings as the gold standard. Osteoarthritic US findings were very common, but no association between the US findings and OHS was observed. The pooled inter-rater reliability (*n* = 65) varied from moderate to excellent (k = 0.538–0.815). When THA findings were used as the gold standard, US detected femoral collum osteophytes with 95% sensitivity, 0% specificity, 81% accuracy, and 85% positive predictive value. Concerning acetabular osteophytes, the respective values were 96%, 0%, 88% and 91%. For the femoral head deformity, they were 92%, 36%, 38% and 83%, and for the effusion 49%, 85%, 58% and 90%, respectively. US provides similar detection of osteophytes as does CR. On femoral head deformity, performance of the US is superior to CR. The inter-rater reliability of the US evaluation varies from moderate to excellent, and no association between US and OHS was observed in this patient cohort.

## Introduction

Hip osteoarthritis (OA) causes significant morbidity, lowers the quality of life, and creates a considerable socioeconomic burden worldwide. As the global life expectancy and the prevalence of obesity is rising, the amount of OA patients will rise in the future^1^. The estimated lifetime risk of symptomatic hip OA is 25% for people who live to age of 85 years^2^. Additionally, approximately 10% lifetime risk of undergoing a total hip arthroplasty (THA) for late-stage hip OA exists^3^. As the treatment of early-stage hip OA is mainly conservative, in late-stage when daily activity is severely compromised, the treatment of choice is THA. Currently, the diagnosis of hip OA consists of patient history, physical examination, and conventional radiography (CR) which is deemed as the primary imaging method of choice in OA. However, a poor association between the CR findings and clinical symptoms in hip OA exists^4^. Ultrasonography (US) is an emerging modality in OA diagnostics. For instance in the knee OA, US has been considered even to outperform CR^5–9^. In the hip joint, US can be used to determine the presence of osteophytes, synovial fluid/synovitis and to evaluate the morphology of the femoral head^10–12^. The only original publication systematically describing US evaluation of hip OA is by Qvistgaard et al., who developed a scoring system for hip OA, assessed the reproducibility of hip US and the correlation to pain^10^. Although hip US offers a non-invasive, widely available, low-cost tool without radiation burden, the literature on hip OA is very scarce. Thus, the objective of this study was to evaluate the US findings on patients with late-stage hip OA scheduled for THA, and to associate the US findings with radiographic and intraoperative findings. Moreover, the inter-observer reliability of the hip US, and association between the US and Oxford Hip Score (OHS) were assessed.

## Results

### The prevalence of US findings and inter-rater reliability

**Figure 1:**
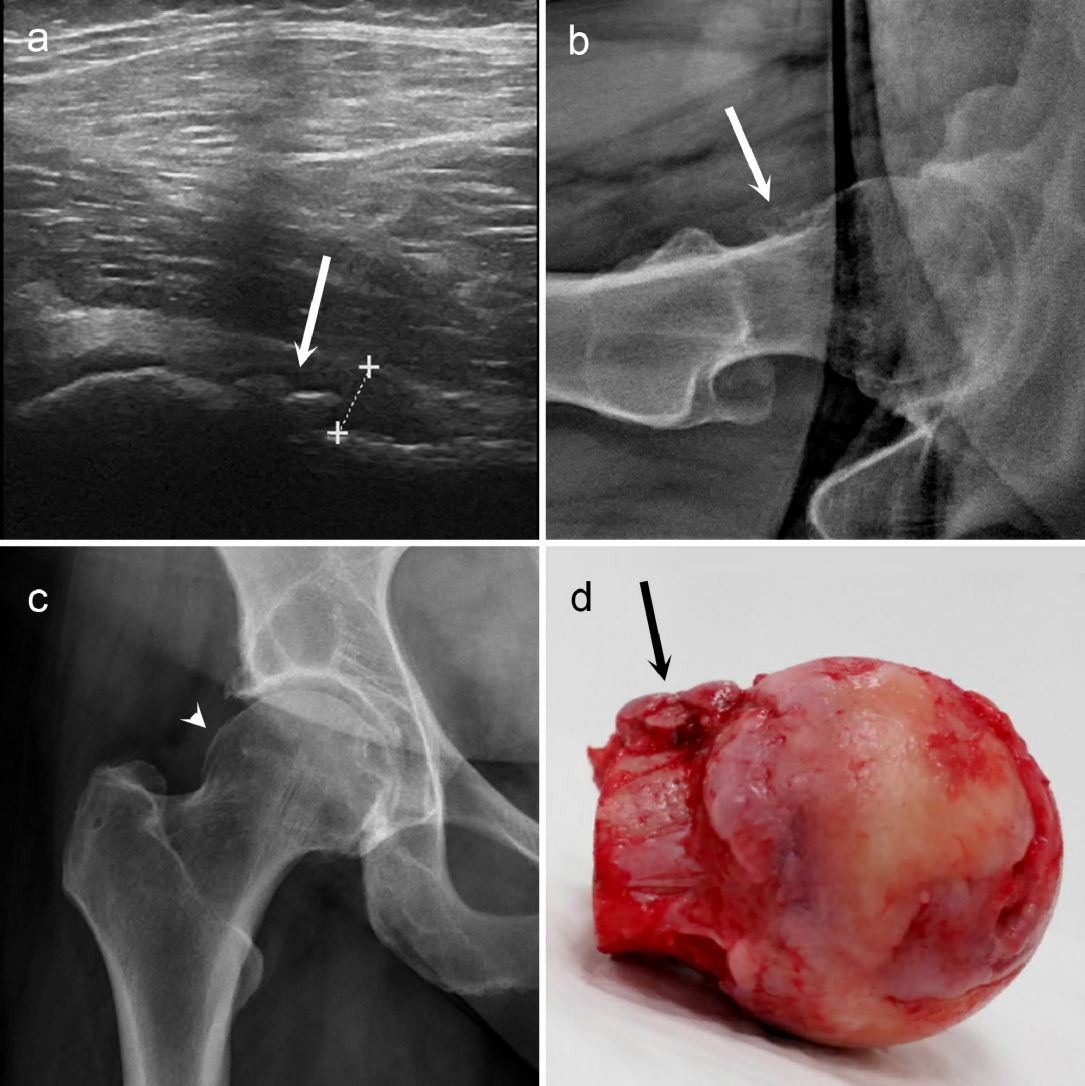
Large femoral collum osteophytes (white arrows) in longitudinal ultrasonographic view (**a**) and lateral conventional radiographic view (**b**). In the antero-posterior radiography (**c**), the same osteophyte is barely visible (white arrowhead). During the total hip arthroplasty, the femoral head was removed, and the corresponding osteophytes (black arrow) are clearly distinguishable at the femoral collum (**d**). In ultrasonography, a small effusion is also observed (measured within the dashed lines).

Osteoarthritic US findings were highly prevalent in this study population: Effusion (≥ 8 mm) was detected in 21/68 (31%) of the cases and the mean thickness was 7.2 mm (SD 2.7 mm, range 4 to 18 mm). In the femoral collum, small osteophytes were observed in 15/68 (22%) and large osteophytes in 51/68 (75%) of the cases. In the anterior acetabulum, small osteophytes were seen in 32/68 (47%) and large osteophytes in 34/68 (50%) of the cases. Concerning the shape of the femoral head, slightly flattened appearance was observed in 46/68 (68%) and distinct deformity in 15/68 (22%) of the cases. Figure 1 demonstrates an example of femoral collum osteophytes in US view, in CR views, and in THA image. No association between the US findings and OHS was observed. Furthermore, neither the CR findings nor the KL grade showed association with the OHS. The US sum score showed weak but significant correlation with KL grades (*r* = 0.223; *p* = 0.034).

Table 1 shows inter-rater reliability between the radiologist and the three independent sonographers. The pooled inter-rater agreement (*n* = 65) varied from moderate to excellent: for the effusion (≥ 8 mm) PABAK was 0.754 (CI 0544–0.891), for the femoral collum osteophytes 0.754 (CI 0.544–0.891), for the acetabular osteophytes 0.815 (CI 0.620–0.931), and for the deformity of the femoral caput 0.538 (CI 0.296–0.729).

**Table 1:** The inter-rater reliability of the hip ultrasonography between the radiologist and the three independent sonographers. Prevalence and bias adjusted kappa (PABAK) with 95% confidence intervals are presented.

	Effusion/synovitis	Osteophytes at femoral collum	Osteophytes at acetabulum	Femoral head deformity
Sonographer 1 (n = 21)	0.810 (0.392–0.977)	0.810 (0.392–0.977)	0.714 (0.273–0.939)	0.619 (0.162–0.891)
Sonographer 2 (n = 22)	0.789 (0.337–0.974)	0.909 (0.543–0.998)	0.909 (0.543–0.998)	0.364 (0.000–0.723)
Sonographer 3 (n = 22)	0.636 (0.194–0.896)	0.545 (0.093–0.844)	0.818 (0.417–0.978)	0.636 (0.194–0.896)

### US findings vs. THA

To evaluate the performance of the US and CR to detect osteoarthritic changes, the macroscopic findings during the THA were used as the gold standard. For the US-detected femoral collum osteophytes, the sensitivity was 95%, the specificity 0%, accuracy 81% and positive predictive value 85%. Concerning acetabular osteophytes, the respective values were 96%, 0%, 88% and 91%. For the deformity of the femoral head they were 92%, 36%, 38% and 83%, respectively. On US-detected effusion, the respective values were 49%, 85%, 58% and 90%. Table 2 summarizes the performance of the US.

**Table 2:** Performance of ultrasonography on detecting osteoarthritic changes of the hip when using the intra-operative findings of total hip arthroplasty as the gold standard.

	TP/N1	Sensitivity, % (95% CI)	TN/N2	Specificity, % (95% CI)	N3	Accuracy, % (95% CI)	Positive predictive value, % (95% CI)	Negative predictive value, % (95% CI)
**Osteophytes**								
Femoral collum	39/41	95 (84–99)	0/7	0 (0–41)	39	81 (67–91)	85 (84–86)	0
Anterior acetabulum	42/44	96 (85–99)	0/4	0 (0–60)	42	88 (75–95)	91 (91–92)	0
**Deformity of the femoral head**	34/37	92 (78–98)	4/11	36 (11–69)	38	79 (65–90)	83 (76–89)	57 (26–84)
**Effusion**	17/35	49 (31–66)	11/13	85 (55–98)	28	58 (43–72)	90 (69–97)	38 (29–48)

TP/N1 = Number of true positives/positive intraoperative findings. TN/N2 = Number of true negatives/negative intraoperative findings.

N3 = Total number of readings concordant with intraoperative findings in 48 hips.

95% CI = 95% confidence interval.

When the performance of CR was evaluated similarly, femoral osteophytes were reported with 90% sensitivity, 14% specificity, 79% accuracy and 86% positive predictive value. For the acetabular osteophytes, the respective values were 98%, 0%, 90% and 92%. Concerning deformity of the femoral head they were 46%, 64%, 50% and 81%, respectively. Table 3 outlines the performance of the CR. When the diagnostic performance of the US and CR were compared using McNemar’s tests, US outperformed CR only on the deformity of the femoral head. Table 4 depicts the diagnostic capacity of the US and CR when the THA findings were used as the gold standard.

**Table 3:** Performance of radiography on detecting osteoarthritic changes of the hip when using the intra-operative findings of total hip arthroplasty as the gold standard.

	TP/N1	Sensitivity, % (95% CI)	TN/N2	Specificity, % (95% CI)	N3	Accuracy, % (95% CI)	Positive predictive value, % (95% CI)	Negative predictive value, % (95% CI)
**Osteophytes**								
Femoral collum	37/41	90 (77–97)	1/7	14 (36–58)	38	79 (65–90)	86 (82–89)	20 (3–66)
Anterior acetabulum	43/44	98 (88–100)	0/4	0 (0 – 6)	43	90 (77–97)	92 (91–92)	0
**Deformity of the femoral head**	17/37	46 (29–63)	7/11	64 (31 – 89)	24	50 (35–65)	81 (64–91)	26 (17–37)

TP/N1 = Number of true positives/positive intraoperative findings. TN/N2 = Number of true negatives/negative intraoperative findings.

N3 = Total number of readings concordant with intraoperative findings in 48 hips.

95% CI = 95% confidence interval.

**Table 4:** Ultrasonography (US) findings versus conventional radiography (CR) findings when intra-operative total hip arthroplasty findings were used as the gold standard.

US finding vs. radiography finding	Total	US+/CR+ N (%)	US+/CR− N (%)	US−/CR+ N (%)	US−/CR− N (%)	P
**Osteophytes**						
Femoral collum	41	35 (85.4)	4 (9.8)	2 (4.9)	0 (0.0)	0.687
Anterior acetabulum	44	41 (93.2)	1 (2.3)	2 (4.5)	0 (0.0)	1.000
**Deformity of the femoral head**	37	16 (43.2)	18 (48.6)	1 (2.7)	2 (5.4)	< 0.001

*US*+ positive in ultrasound, *US− *negative in ultrasound, *R*+ positive in radiography, *R− *negative in radiography.

## Discussion

**Figure 2:**
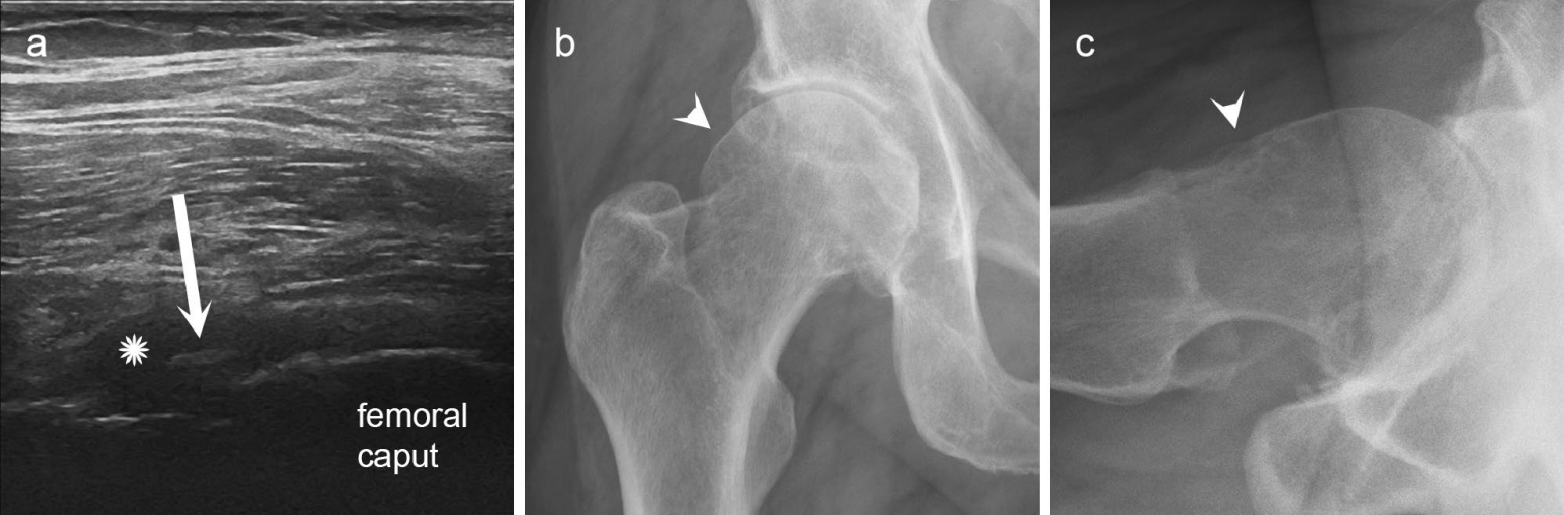
An example case where ultrasonography (US) detected osteophyte and radiography did not. In longitudinal US view of the hip joint (**a**), a large femoral collum osteophyte is observed (white arrow), whereas on anteroposterior (**b**) and lateral conventional radiographic views (**c**) only doubtful bony prominence but no definite osteophytes are seen (white arrowheads). Additionally, the US demonstrates an effusion (white asterisk) of the hip joint.

**Figure 3:**
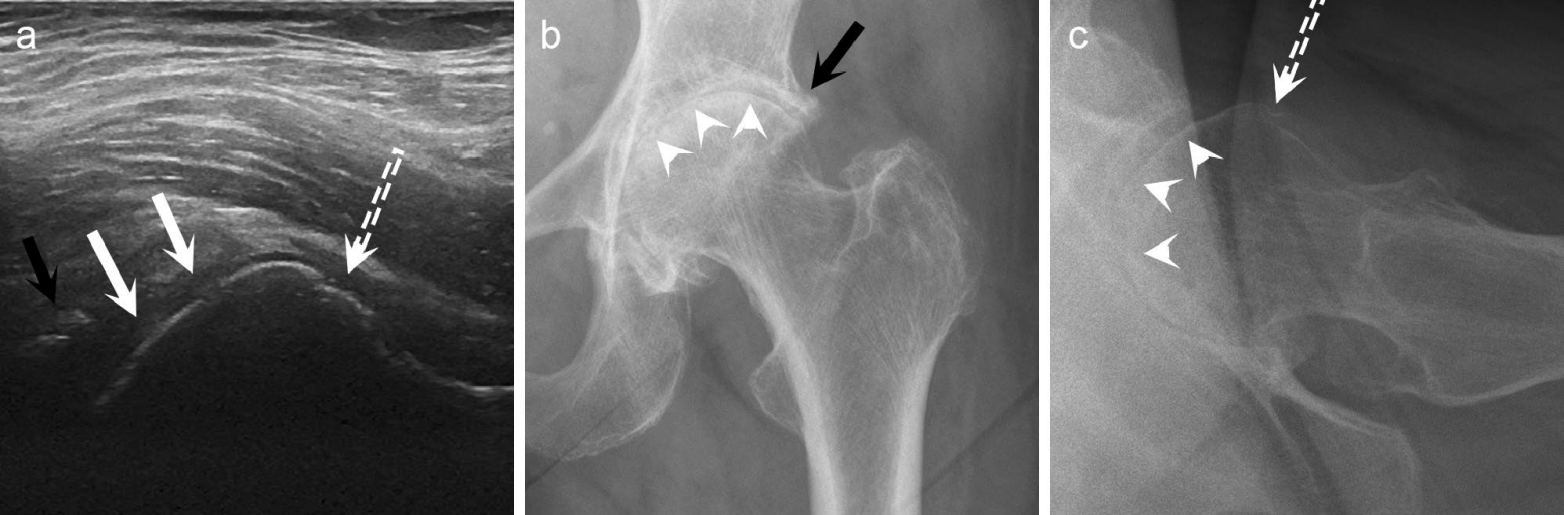
Ultrasonography (US) detects deformity of the femoral head better than conventional radiography (CR). In longitudinal US view of the hip joint (**a**), marked deformity of the femoral head is seen (white arrows), whereas on anteroposterior (**b**) and lateral CR views (**c**) the femoral head has preserved its normal ball-like appearance (white arrowheads). Additionally, a large femoral acetabular osteophyte (black arrow) is observed on US (**a**) and anteroposterior CR (**b**). Furthermore, femoral collum osteophyte (dashed white arrow) is seen on US (**a**) and on lateral CR (**c**).

In this study, we compared the US and CR of findings of the late-stage hip OA with the intraoperative findings of the THA. Results indicate that the US is as good as CR in detecting the osteophytes of femoral collum and anterosuperior acetabulum. Figure 2 shows an example of a large femoral collum osteophyte visible on US but not on CR. Concerning the morphology of the femoral head, US slightly outperformed CR (Fig. 3). It is noteworthy that the previous literature on US of the hip OA is very scarce, and that macroscopic correlation studies—applying arthroscopy or open surgery as the gold standard—were completely lacking. In the year 2006, Qvistgaard et al. introduced the US grading system of hip OA, which was also applied in this study. The authors reported only a weak correlation between KL scores and two osseous US scores: KL grade vs. collum osteophytes (*r* = 0.26, *p* = 0.017) and KL grade vs. femoral head deformity (*r* = 0.24, *p* = 0.03)^10^. In a more recent population study including 311 subjects of 63 years of age, Abraham et al. found that US is more sensitive to detect hip OA than CR; however, the authors studied only the hip osteophytes and femoral head deformity^18^. Even though our results suggest high sensitivity for US to detect osteophytes, the absence of normal findings pushes the specificity to zero. Interestingly, every US feature assessed in our study showed excellent positive predictive value (83–91%) highlighting the common feature of US examination in the OA applications; positive findings are trustworthy but negative finding does not rule out pathology. This highly contradicts the expert opinion by European Society of Musculoskeletal Radiology from the year 2012, which does not recommend US for hip osteoarthritis diagnostics^19^.

Since no relevant Doppler signal was acquired in the first 20 patients, we only assessed the capsular bulging on the femoral collum. Accordingly, we observed poor sensitivity (49%), but good specificity (85%) and positive predictive value (90%), for the detecting the hip effusion (≥ 8 mm) when the corresponding THA finding was used as the gold standard. Previously, Qvistgaard et al. (2006) reported virtually no association between the US-detected effusion and joint aspiration; however, their gold standard was not as reliable as ours^10^. The inefficiency of the Doppler imaging remains somewhat disconcerting and may be related to the US device that we used, since in a small sample of 24 hips undergoing THA, Walther et al. found that Power Doppler US is a reliable tool to detect synovitis; they observed a significant correlation between the US and histopathologic findings^20^.

We found no association between the US findings and the OHS. On the contrary to our study, Qvistgaard et al. found association between US-detected hip OA and the patient’s pain at rest and on walking^10^. In general, the OA’s association with symptoms remains debatable. In a large study including over 5000 subjects, Kim et al. established that hip pain was not present in many hips with radiographic OA, and many painful hips did not show radiographic hip OA^4^. On MRI, Kumar et al. suggested that acetabular cartilage defects, bone marrow lesions and subchondral cysts were associated with hip pain and dysfunction. However, the authors observed no association between radiographic hip OA and pain, which supports our findings here^21^.

Only two previous studies assessing the reproducibility of US of osteoarthritic hip joint exist. With 100 patients, Qvistgaard et al. reported that the intra-rater reliability (kappa) was 0.75 for osteophytes, 0.69 for femoral head deformity, 0.58 for effusion and 0.55 for synovitis. The corresponding inter-rater reliability was 0.49, 0.35, 0.38 and 0.43, respectively. As a minor weakness, only image re-interpretation was performed, not a second US scan^10^. Later, Abraham et al. (2014) found that inter-rater reliability was only moderate (kappa 0.47) for femoral collum osteophytes and femoral head deformity^18^. Our results are in line with these previous studies with the inter-rater agreement being between moderate and almost perfect (PABAK 0.538–0.815). Our slightly better reliability values may be due to the technical development of the US devices and the corresponding spatial resolution and contrast.

Several limitations exist in our study. First, the high prevalence of OA findings induces bias to this study, which is reflected mostly as the very low specificity obtained by the US evaluation since almost every patient had a positive finding. Second, the time from the US assessment to the TKA operation slightly varied and the amount of effusion could have changed. Third, the cartilage of the femoral head was not assessed on US, leaving no possibility for correlation with joint-space narrowing on CR; however, the diagnostic view on US to the femoral head cartilage is presumably insufficient. Fourth, the high BMI of few patients weakened the diagnostic US window allowing misinterpretation of US findings. Fifth, the rather large number of operating orthopedic surgeons may have created variation to the documentation of TKA findings; therefore, the grading was kept as effortless as possible. Additionally, the surgeons were not blinded to the CR images, allowing biased classification of the intraoperative findings.

In conclusion, on severe hip OA, US provides similar detection of osteophytes as does CR. On femoral head deformity, the performance of the US is superior to CR. The inter-rater reliability of the US evaluation varies from moderate to excellent, and no association with Oxford Hip Score exists.

## Methods

### Patients

Sixty-six patients scheduled for THA for late-stage OA of the hip were enrolled consecutively in this study during November 2017 and March 2018. The mean age was 67.6 years (range 50 to 88 years) and 38% were males. Two patients had bilateral THA, thus, in total 68 hips were included in this study initially. One patient failed to report the OHS, and the intraoperative findings were available on 48 hips. Written informed consent was obtained from every patient. This prospective diagnostic study (level of evidence: III) was carried out in accordance with the Declaration of Helsinki and approved by the Ethical Committee of Northern Finland Health Care District, Oulu University Hospital (number 106/2017).

### Ultrasonography

US imaging was conducted using a clinical US device (LOGIQ S7, GE Healthcare, Milwaukee, WI, USA) with 15 MHz linear transducer ML6–15. If the deep location of the hip joint hindered the visibility, the 5 MHz convex transducer C1-5-D was applied to depict the anatomy. B-mode imaging settings were kept constant for each subject and the focus was set at the level of region of interest. US of the hip was performed by a single radiologist with four years of experience. Second ultrasonographic assessment was performed by three independent sonographers—all with more than five years of experience—on 65 hips to evaluate the inter-reader reliability. All observers were blinded to the clinical and radiographic findings.

The hip was scanned with patient in supine position with toes slightly turned outwards (eversion) in anterior-longitudinal plane parallel to the femoral neck to assess effusion, osteophytes, appearance of femoral head. The probe was moved from a medial to lateral direction to obtain the optimal image. Doppler imaging yielded no signal on the first 20 patients, so it was not further utilized in this study. The US findings were graded by each observer according to Qvistgaard et al.^10^. In the literature, the threshold for hip effusion varies between 7 and 9 mm^13–16^; thus thickness of fluid or capsular bulge of at least 8 mm in collum/caput interface was defined as effusion in our study. The presence and size of osteophytes was evaluated anteriorly at the femoral collum and anterior acetabulum as follows: Grade 0 = no osteophyte, Grade 1 = small osteophyte, Grade 2 = large osteophyte. The contour of the femoral head was classified Grade 0 = normal, Grade 1 = slightly flattened, or Grade 2 = clearly deformed.

### Radiography

One to 95 weeks (mean 20 weeks) before the US study, the patients underwent hip standard anteroposterior hip CR with an addition of a cross-table lateral view projection of the symptomatic side. In the anteroposterior view, patient is in a supine position and lower extremities were internally rotated to 15°–20°. The image was centered to the upper part of the symphysis pubis. In the cross-table lateral view the patient was in supine position, with hip in 15°–20° internal rotation, and the contralateral hip and knee in 90° flexion to exclude the unaffected lower extremity from the image. The projection was toward groin region at 45° of incidence parallel to the longitudinal axis of the femur.

The CR of the hips were assessed by one radiologist with five years of experience for osteophytes, joint space narrowing, appearance of the femoral caput and Kellgren-Lawrence (KL) grades. Osteophytes were graded at femoral collum and superior anterolateral acetabulum as follows: Grade 0 = no osteophyte, Grade 1 = marginal/small osteophyte, Grade 2 = a definite osteophyte. Joint space was defined either normal or narrowed. The contour of the femoral head classified either Grade 0 = normal, Grade 1 = slightly irregular, or Grade 2 = clearly deformed. Ultimately, the total KL grade was given for the hip joint. The reader was blinded to clinical and US findings.

### Total hip arthroplasty findings

The THA operation was performed 3 to 39 days (mean 15 days) after the US evaluations by eight orthopedic surgeons with at least 10 years of THA experience. The surgeons were blinded to the US findings, but not to clinical history and CR findings. Routine posterior approach for THA was conducted and the surgical findings were collected as follows: effusion (no, yes), anterior osteophytes on femoral collum (no, yes) and on acetabulum (no, yes), and the deformity of the femoral caput (no, yes). The intraoperative grading was kept simple due to several different surgeons performing the THAs.

### Statistical analysis

Owing to the rather small sample size and distributions of US findings, cut-offs were applied to create dichotomous score on certain variables: both US-detected and CR-detected osteophytes were categorized as non-significant (Grade 0) or significant (Grades 1 and 2); similarly femoral head deformity was grade as non-significant (Grade 0) or significant (Grades 1 and 2) on both imaging modalities. After dichotomizing, an US sum score ranging from zero to four was created. Data of US and CR findings are given as numbers of true positive and negative findings according to intraoperative findings. Sensitivity, specificity, accuracy, positive predictive value, and negative predictive value with their 95% confidence intervals were calculated for each finding. Confidence intervals for sensitivity, specificity and accuracy were calculated using Clopper-Pearson confidence interval; for predictive values the standard logit confidence intervals were applied^17^. The sensitivities between US and radiography were compared within positive intraoperative findings using Mc-Nemar’s test. To evaluate the associations of US and CR findings with OHS, Mann–Whitney and Kruskal–Wallis methods were used. For correlation analyses, Spearman’s rho was calculated. P-value < 0.05 was considered as statistically significant. To evaluate inter-reader agreement, prevalence-adjusted and bias-adjusted kappa (PABAK) was applied with the following interpretation: PABAK values ≤ 0 indicated no agreement, 0.01–0.20 indicated none to slight agreement, 0.21–0.40 indicated fair agreement, 0.41–0.60 indicated moderate agreement, 0.61–0.80 indicated substantial agreement, and 0.81–1.00 indicated excellent agreement. SPSS 24.0 and R 3.5.2 were used for data analysis.

## Data Availability

The datasets generated during and/or analysed during the current study are available from the corresponding author on reasonable request.

## References

[1] Osteoarthritis Research Society International (2016). Osteoarthritis: a serious disease. Osteoarthr. Res. Soc. Int..

[2] Murphy LB (2010). One in four people may develop symptomatic hip osteoarthritis in his or her lifetime. Osteoarthr. Cartil..

[3] Culliford DJ (2012). The lifetime risk of total hip and knee arthroplasty: results from the UK general practice research database. Osteoarthr. Cartil..

[4] Kim C (2015). Association of hip pain with radiographic evidence of hip osteoarthritis: diagnostic test study. BMJ.

[5] Koski JM (2016). Atlas-based knee osteophyte assessment with ultrasonography and radiography: relationship to arthroscopic degeneration of articular cartilage. Scand. J. Rheumatol..

[6] Okano T (2016). Ultrasonographic evaluation of joint damage in knee osteoarthritis: feature-specific comparisons with conventional radiography. Rheumatology.

[7] Podlipská J (2016). Comparison of diagnostic performance of semi-quantitative knee ultrasound and knee radiography with MRI: Oulu Knee osteoarthritis study. Sci. Rep..

[8] Serban O (2016). Pain in bilateral knee osteoarthritis: correlations between clinical examination, radiological, and ultrasonographical findings. Med. Ultrason..

[9] Nevalainen MT (2018). Ultrasonography of the late-stage knee osteoarthritis prior to total knee arthroplasty: comparison of the ultrasonographic, radiographic and intra-operative findings. Sci. Rep..

[10] Qvistgaard E, Torp-Pedersen S, Christensen R, Bliddal H (2006). Reproducibility and inter-reader agreement of a scoring system for ultrasound evaluation of hip osteoarthritis. Ann. Rheum. Dis..

[11] Nestorova R (2012). Ultrasonography of the hip. Med. Ultrason..

[12] Sudula SN (2016). Imaging the hip joint in osteoarthritis: a place for ultrasound?. Ultrasound..

[13] Koski JM, Anttila PJ, Isomäki HA (1989). Ultrasonography of the adult hip joint. Scand. J. Rheumatol..

[14] Koski JM, Anttila P, Hämäläinen M, Isomäki H (1990). Hip joint ultrasonography: correlation with intra-articular effusion and synovitis. Br. J. Rheumatol..

[15] Sada PN, Rajan P, Jeyaseelan L, Washburn MC (1994). Standards for ultrasonographic measurements of the hip joint in Indian adults. Skeletal Radiol..

[16] Schmidt WA, Schmidt H, Schicke B, Gromnica-Ihle E (2004). Standard reference values for musculoskeletal ultrasonography. Ann. Rheum. Dis..

[17] Mercaldo ND, Lau KF, Zhou XH (2007). Confidence intervals for predictive values witan emphasis to case-control studies. Stat Med..

[18] Abraham AM (2014). Population prevalence of ultrasound features of osteoarthritis in the hand, knee and hip at age 63 years: the Newcastle thousand families birth cohort. BMC Musculoskelet. Disord..

[19] Klauser AS (2012). Clinical indications for musculoskeletal ultrasound: a Delphi-based consensus paper of the European Society of Musculoskeletal Radiology. Eur. Radiol..

[20] Walther M (2002). Synovial tissue of the hip at power Doppler US: correlation between vascularity and power Doppler US signal. Radiology.

[21] Kumar D (2013). Association of cartilage defects, and other MRI findings with pain and function in individuals with mild-moderate radiographic hip osteoarthritis and controls. Osteoarthr. Cartil..

